# Assessing the impact of global climate changes on irrigated wheat yields and water requirements in a semi-arid environment of Morocco

**DOI:** 10.1038/s41598-019-55251-2

**Published:** 2019-12-16

**Authors:** Elhoussaine Bouras, Lionel Jarlan, Said Khabba, Salah Er-Raki, Alain Dezetter, Fathallah Sghir, Yves Tramblay

**Affiliations:** 1 LP2M2E, Département de Physique Appliquée, Faculté des Sciences et Techniques, Université Cadi Ayyad, (UCAM), Marrakech, Morocco; 2 Centre d’Etudes Spatiales de la BIOsphère (CESBIO), Institut de Recherche pour le Développement (IRD), Toulouse, France; 3 Joint International Laboratory TREMA, Université Cadi Ayyad (UCAM), Marrakech, Morocco; 4 Center for Remote Sensing Applications (CRSA), University Mohammed VI Polytechnic (UM6P), Benguerir, Morocco; 5 LMME, Faculté des Sciences Semalia (FSS), Université Cadi Ayyad (UCAM), Marrakech, Morocco; 6 Laboratoire HydroSciences Montpellier (HSM), Montpellier, France; 7 Office Régional de Mise en Valeur Agricole du Haouz (ORMVAH), Marrakech, Morocco

**Keywords:** Climate-change impacts, Hydrology

## Abstract

The present work aims to quantify the impact of climate change (CC) on the grain yields of irrigated cereals and their water requirements in the Tensift region of Morocco. The Med-CORDEX (MEDiterranean COordinated Regional Climate Downscaling EXperiment) ensemble runs under scenarios RCP4.5 (Representative Concentration Pathway) and RCP8.5 are first evaluated and disaggregated using the quantile-quantile approach. The impact of CC on the duration of the main wheat phenological stages based on the degree-day approach is then analyzed. The results show that the rise in air temperature causes a shortening of the development cycle of up to 50 days. The impacts of rising temperature and changes in precipitation on wheat yields are next evaluated, based on the AquaCrop model, both with and without taking into account the fertilizing effect of CO_2_. As expected, optimal wheat yields will decrease on the order of 7 to 30% if CO_2_ concentration rise is not considered. The fertilizing effect of CO_2_ can counterbalance yield losses, since optimal yields could increase by 7% and 13% respectively at mid-century for the RCP4.5 and RCP8.5 scenarios. Finally, water requirements are expected to decrease by 13 to 42%, mainly in response to the shortening of the cycle. This decrease is associated with a change in temporal patterns, with the requirement peak coming two months earlier than under current conditions.

## Introduction

Climate changes (CC), mainly attributed to the human-induced increase of greenhouse gases, e.g., carbon dioxide CO_2_, are expected to cause global warming in certain regions over the next century^1^. In particular, the Mediterranean area has been identified as a hot spot of climate change^2^. As a result, numerous key economic sectors, including agriculture, could be drastically impacted. In Morocco, winter cereals occupy more than 55% of the country’s agricultural areas, common and durum wheat account for about 75%^3^. Although it represents only 19% of arable land, irrigated agriculture contributes 45% of added agricultural value. It consumes about 83% of the available resources^4^, but could be the main lever of water saving in the region as legislation remains permissive and irrigation efficiency is low. Climate change is expected to lead to an even stronger production variability leading than today in turn to price volatility^5^. Given this context, it becomes important to analyze the impact of future climate changes on wheat yield and irrigation water requirements in this region.

Higher atmospheric CO_2_ concentration, and changes in temperature and rainfall may increase or decrease crop yield, and the net effect of CC on crop yield depends on the interaction between these various factors. Plant production and water-use efficiency are known to increase at higher atmospheric CO_2_ concentrations, in particular for C3 plants such as wheat. This is due to higher rates of photosynthesis^6^ and an improved response to stress related to a reduced stomatal closure that better regulates plant transpiration^7^. On the other hand, higher atmospheric CO_2_ can negatively affect grain quality by reducing the concentrations of plant nutrients^8^. Moreover, increased temperature can negatively impact plant production because of the heat stress^9^. This is particularly the case if it occurs at sensitive phenological stages, e.g., pollination for wheat^10^ and because it reduces the length of the crop season, leading to less radiation intercepted by the plants^11^. Another effect of higher temperatures is an increase in plant water demand due to increased transpiration^12^. Obviously, a smaller rainfall during the wheat development cycle at emergence, tillering^13^ or during the grain-filling^14^ stages can drastically affect yield, especially for rainfed crops, owing to a deficit of crop water.

Several studies have employed crop models to study the impact of climate changes on agricultural production, e.g., Ludwig and Asseng^15^; Yang *et al*.^16^ among several others. Considering temperature increase and rainfall changes only, lower yields must inevitably be projected. By contrast, most of the simulation studies carried out on semi-arid Mediterranean environments suggest that fertilizing effects could offset the negative impacts of higher temperatures, resulting in increased yields^15,17,18^ and that this positive impact could be more pronounced in warm and dry locations^19^. Concerning water requirements, Saadi *et al*.^17^ demonstrate that under optimal conditions, the net irrigation requirements could decrease by 11% at the 2050 horizon in the southern Mediterranean region. Nevertheless, most studies instead suggest an increase in water requirements, Lovelli *et al*.^20^ explained the rise of evaporative demand associated with rising temperature^21^ could not be compensated by reductions in phenological stages and partial stomatal closure^22^. Other studies attempt additionally to take into account the change in land-use associated with the major agricultural transformation that faces the Mediterranean regions (intensification, conversion to cash crops, and so on). Valverde *et al*.^23^ and Rodriguez-Diaz *et al*.^24^ in the Iberian Peninsula, both found a substantial increase in water requirements but without considering the potential reduction in the duration of the phenological stages. Likewise, the recent work of Fader *et al*.^25^ also suggests a significant increase in gross irrigation that could reach 74% in the more extreme scenarios. To our knowledge, no studies has been designed for studying the potential changes in the seasonal irrigation patterns in south Mediterranean while a different timing of the irrigation season induced by changes in the phenological stages duration may have significant consequences for water management.

This literature review demonstrates the complex interactions between the various manifestations of climate change, and shows that the simulated impacts of their combined effects on yields and water requirements depend strongly on local conditions and also on the various process representations used in the modeling tools. In particular, the accuracy of the impact projections depends critically on the representation of the fertilization effect within the crop models^26^. Crop models used in impact assessment studies simulate the effects of elevated CO_2_ on growth and yield by a variety of methods (reviewed by Tubiello and Ewert^27^). Some concerns have been expressed^26,28,29^ regarding the parameterizations that have been developed from earlier studies implemented in greenhouses. More recent Free Air Carbon dioxide Enrichment (FACE) experiments tend to demonstrate that fertilizing effects projected from enclosure studies may overestimate the fertilizing effect by up to 50%^26^. In order to address this large uncertainty, impact projection studies of climate change on crop production are now usually based on two experiments: one taking into account the fertilizing effect and another that does not^25^, in addition to considering several CO_2_ emission scenarios.

The objective of the present work is to study the impact of climate change, including rising atmospheric CO_2_ concentration and temperature, and changes in rainfall, on the optimal grain yield and water requirements of irrigated wheat in the Tensift region (Morocco). The optimal grain yield refers to a yield obtained under optimal irrigation scheduling and optimal fertilizer rates. To this objective, we considered two scenarios (RCP4.5 and RCP8.5) and two horizons (2041–2060 and 2081–2100). In order to assess the impact on yields, we choose the AquaCrop model^30^ because (1) it was calibrated on wheat grown in the study region during a previous investigation^31^; (2) it has already been used in impact projection studies^32–35^; (3) the CO_2_ fertilizing effect has been parameterized based on FACE experiment results^36,37^.

## Results

### Temperature and precipitation trends

**Figure 1:**
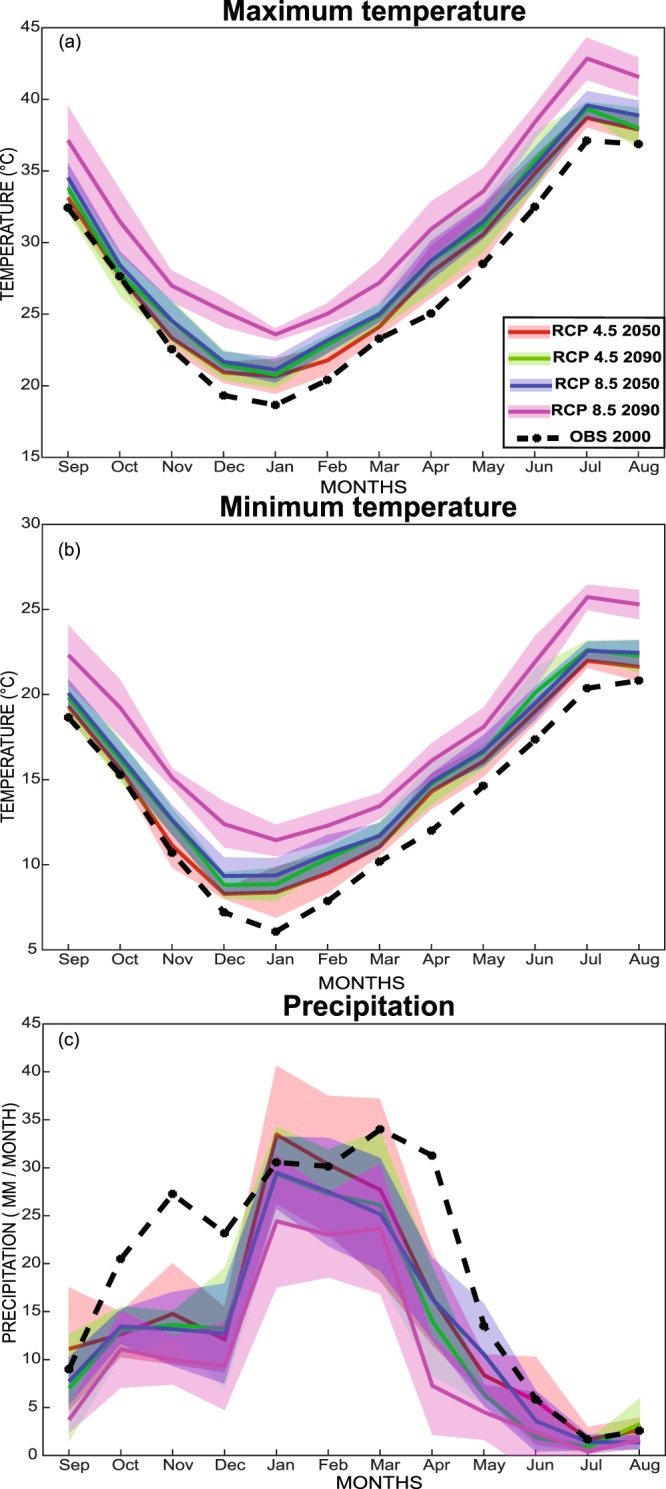
Projected maximum temperature (**a**), minimum temperature (**b**) and precipitation (**c**) according to RCP 4.5 and RCP8.5 in 2050 and 2090. The line is the models mean and the colored section corresponds to Mean ± SD standard deviation of the 5 RCM models.

Future changes in temperature and precipitation under the RCP4.5 and RCP8.5 scenarios between the historical period 1991‒2010 and the two time horizons 2041–2060 (referred to hereafter as 2050) and 2081–2100 (hereafter 2090) were evaluated. For all scenarios, there is a systematic increase in maximum and minimum temperature ranging from 1 °C (RCP4.5 by 2050) to 6 °C (RCP8.5 by 2090) (Fig. 1a,b). Even considering the models’ overall spread, there is no ambiguity concerning the sign of the change, which remains positive apart from RCP4.5 during September and October. The higher increase is logically observed for the most distant horizons and for the most pessimistic emission scenarios (Fig. 1a,b). Interestingly, the seasonal cycle of minimum and maximum temperature change is fairly marked, with a much stronger increase during the winter months. For instance, the average difference between the RCP4.5 2050 minimum temperature and observed values during December-January-February is 1.8 °C, while this difference for March-April-May is only 1.2 °C. These increased temperature will also affect reference evapotranspiration ($E{T}_{0}$). In our study, annual $E{T}_{0}$ is expected to increase by 4.3, 7.5, 7.8 and 9.2% respectively under RCP4.5 by 2050, RCP4.5 by 2090, RCP8.5 by 2050 and RCP8.5 by 2090 (not shown).

A decrease in precipitation (from the mean of the model ensemble) is expected for mostly all scenarios (Fig. 1c) and period. This lower precipitation is particularly prominent in spring (March and April) and winter (October to December) even when considering the model ensemble’s spread. The drop may reach 12 mm and 10 mm per month in spring, for instance, under RCP8.5 in 2050 and 2090 respectively. This corresponds to 38% and 45% of the monthly rainfall.

### Impact of CC on the duration of phenological stages

The impact of CC on the total duration of the cycle is first quantified starting by the variation of the cumulative degree days (CDD), a classic method for predicting wheat development^38^. For the historical period (1991–2010), is equal to 2372 °C for the observations at the meteorological station of Marrakech; an increase is observed for all scenarios and horizons ranging from 3% (RCP4.5 2050) to 30% (RCP8.5 2090) (Fig. S1). For information, no specific effect of CO_2_ has been observed on crop cycle duration, either from modeling studies^39,40^ or from FACE experiments^41^, implying that temperature rise explains the change in phenology.

**Figure 2:**
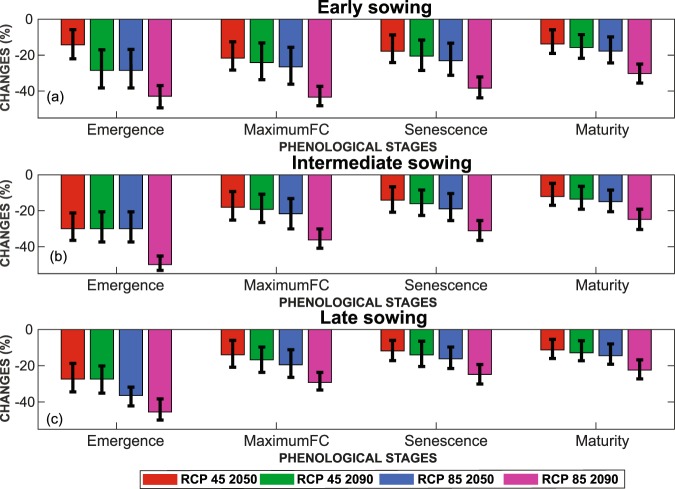
Reduction of the duration of the phenological stages of wheat (in %) for different climatic scenarios and horizon: (**a**) early sowing (**b**) intermediate sowing and (**c**) late sowing. The error bars correspond to Mean ± SD of the 5 RCM models.

In the AquaCrop model, the phenological stages are emergence, maximum canopy cover, senescence and maturity, the time from sowing to maturity corresponds to the length of the crop season (LCS). Actual phenological stage’s durations are presented in Table 1. The results show a systematic reduction of the LCS from 10 to 32% (Fig. 2). Small differences are observed between the sowing dates as, for instance, the early-season LCS could be decreased by 11% (about 17 days) for RCP4.5 at 2050 and a 10% reduction is expected under the same conditions for late sowing (about 13 days). The same trends but with stronger reductions are observed for the other scenarios and horizons. For the more extreme scenario at the end of the century (RCP8.5 2090), the difference is higher but remains limited (32% decrease for early sowing versus 26% decrease for late sowing). Considering the model ensemble’s spread, the uncertainty can be high, in particular for RCP4.5 in 2050, but the phenological stage durations are always predicted to decrease whatever the scenarios and horizons.

**Table 1:** Actual (year 2000) duration of the phenological stages for all sowing dates.

Stage\Sowing date	Emergence (days)	Maximum canopy Cover (days)	Start of senescence (days)	Maturity* (days)
Early	7	83	112	152
Intermediate	9	83	106	141
Late	11	72	93	125

^*^Corresponds to the length of the crop season (LCS).

More interestingly, despite a decrease in the total LCS of a similar order of magnitude for all sowing dates, the phenological stages are affected in a different way. The greater temperature rise expected in the winter months primarily affects the emergence stage for late sowing: a 14% reduction is expected for RCP4.5 2050 for early sowing while it could reach 27% for late sowing. By contrast, the early sowing cycle is mainly impacted during the period of maximum canopy cover, which will drop by 22% for RCP4.5 2050 versus a 14% decrease for late sowing.

### Impact of CC on optimal grain yields of wheat

As a preliminary step, the AquaCrop model driven by meteorological observations from the historical period (1991–2010) has been used to compute the baseline optimum grain yields, which are equal to 7.5, 6.2 and 5.4 tons/ha for early, intermediate and late sowing, respectively.

#### Wheat yield under CO_2_-only change (“CO_2_” experiment)

**Figure 3:**
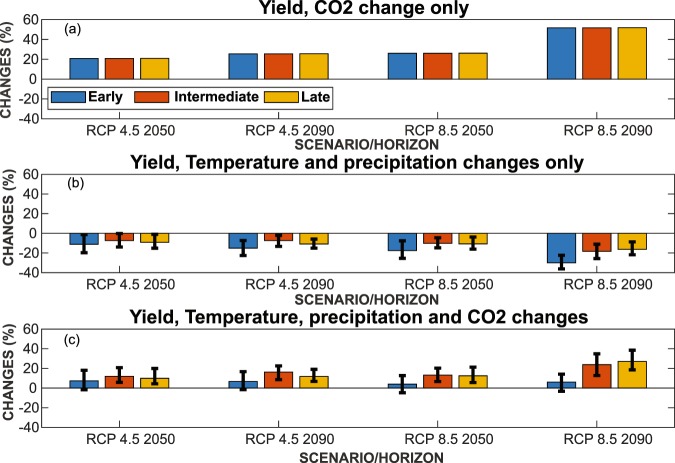
Relative change in yields for early, intermediate and late sowing date corresponding to: (**a**) CO_2_ change only “CO_2_” experiment, (**b**) Temperature and precipitation changes only “CC” experiment and (**c**) temperature, precipitation and CO_2_ changes “CCO2” experiment. The error bars correspond to Mean ± SD of the 5 RCM models.

Experiments that considered only the rise of the CO_2_ concentration predicted significant increases of wheat yield under all scenarios for all time periods (Fig. 3a) because of higher rates of photosynthesis leading to higher plant production and more efficient use of water^6,7^. Around the year 2050 wheat yields could rise between 21 to 26% for RCP4.5 and RCP8.5 respectively. Around the year 2090, yields are expected to increase by as much as 52% for all sowing dates.

#### Wheat yield under temperature and precipitation changes only (“CC” experiment)

When changes in temperature and precipitation only are taken into account, the optimal yields tend to decrease for all sowing dates and all RCPs scenarios and horizons (Fig. 3b). A shorter LCS related to warmer temperature reduces the cumulative intercepted radiation, and, *in fine*, the biomass and grain yields^11,42^. For example, in 2050 for RCP4.5 and RCP8.5, projected yield decreases are 11 and 18% for early sowing associated with a decrease in LCS of 12 and 17%. Similarly, for RCP4.5 in 2090, yields could be reduced by 15, 7 and 11% for early, intermediate and late sowing respectively, under an increase in yearly average temperature of 2.3 °C. Obviously, a more drastic drop is expected in 2090 for the RCP8.5 scenario but with the same trends with respect to the sowing date: for early sowing, a 30% loss of yield due to an increase in temperature of 4.9 °C is projected (associated with a 30% decrease in LCS). The uncertainties of changes in yield for “CC” experiments are lower for intermediate sowing compared to other sowing dates, especially for RCP8.5 2050. For late and intermediate sowing, yield reduction is smaller (9 and 11%) than for early sowing. This is probably because of an already warmer “baseline” temperature during the growth cycle and also because the reduction of the emergence stage for this sowing dates probably has less impact on yields than the larger reduction in the phenological stages related to the maximum canopy cover, which could affect early-sowing wheat. Indeed, Jarlan *et al*.^43^ found a negative correlation between yields and temperature in February and at the end of the cycle in March, corresponding to the high temperatures generally prevailing during the grain-filling stage in early spring. This could explain the larger drop in yield for early sowing because the grain-filling phase would coincide with the months of February and March. Pre- and post-anthesis high temperatures and heating are known to have huge impacts upon wheat growth, since heat stress reduces the photosynthetic efficiency of crops^44^. In particular, Wardlaw and Dunstone^45^ have observed that photosynthesis is optimal for wheat at temperatures between 20 and 30 °C and decreases steeply at temperatures above 30 °C. High temperatures (above 30 °C) are particularly harmful for yields of all crops during the pollination phase^46^. Interestingly enough, this effect is represented in AquaCrop. In our study, the 30 °C threshold is never reached for none of the scenarios testedbut the corruption of extremes values by the Q-Q approach as underlined previously precludes from drawing any definitive conclusion on this point.

#### Wheat yields under temperature, precipitation and CO_2_ changes (“CCO2” experiment)

With a dual increase of temperature and of atmospheric CO_2_ concentration caused by CC, optimal grain yields should rise for all RCPs scenarios and horizons (Fig. 3c). This means that, based on our simulation study, the fertilizing effect might be able to offset the yield loss. In 2050 yield could increase by 7 and 12% for early and intermediate sowing, depending on the RCP scenario. And in 2090 maximum increases in yields are expected for late sowing under RCP8.5 (27%). Taking into account the ensemble’s spread, the sign of the change appears uncertain for early-sowing wheat, which combines a moderate expected average increase of wheat yields and a high uncertainty.

### Impact of CC on water requirements and water productivity

In order to study wheat’s water requirements (including both irrigation and precipitation) and water productivity, we examined the anticipated combined effects of the potential increase in evaporative demand and shortening of the phenological cycle on future water requirements and productivity.

#### Impact of CC on water requirements (WR)

Decrease in cumulative evaporative demand (computed from the Hargreaves formula) associated with a shorter growing season, leads to a decrease in transpiration and evaporation (Fig. S2). Current seasonal cumulative evapotranspiration for early, intermediate and late sowings are 353 ($E$ = 72 mm $Tr$ = 281 mm), 382 (83, 299) and 404 mm (92, 312 mm) respectively. The pa rtition of evapotranspiration is in accordance with the measured values in the region, showing a do minant trans piration^47–51^. In 2050, according to RCP4.5, transpiration is expected to decrease by 57, 48 and 38 mm (20, 16, and 12%) and evaporation also decreases by 16, 9 and 16 mm (22, 11, and 17%) for early, intermediate and late sowing respectively. This corresponds to an evapotranspiration drop of 21, 15 and 13% for the three sowing dates. Also, according to RCP8.5 in 2090, the decrease in evapotranspiration reaches 43% for early sowing. This decrease in evapotranspiration may decrease WR for the various climate scenarios and horizons (Fig. S2). The actual WRs are 353, 382 and 404 mm for early, intermediate and late sowing respectively. These values are consistent with those obtained by Hadria *et al*.^52^, Kharrou *et al*.^53^ and Toumi *et al*.^31^ for wheat in the same region of study. WR was then projected for the CO_2_ and climate change scenarios. Results show a systematic decrease of WR independent of the sowing date and of the scenarios and horizons, indicating that the joint shortening of the LCS and the improved stomatal regulation associated with rising temperature and CO_2_ are able to counterbalance the increase in evaporative demand.

**Figure 4:**
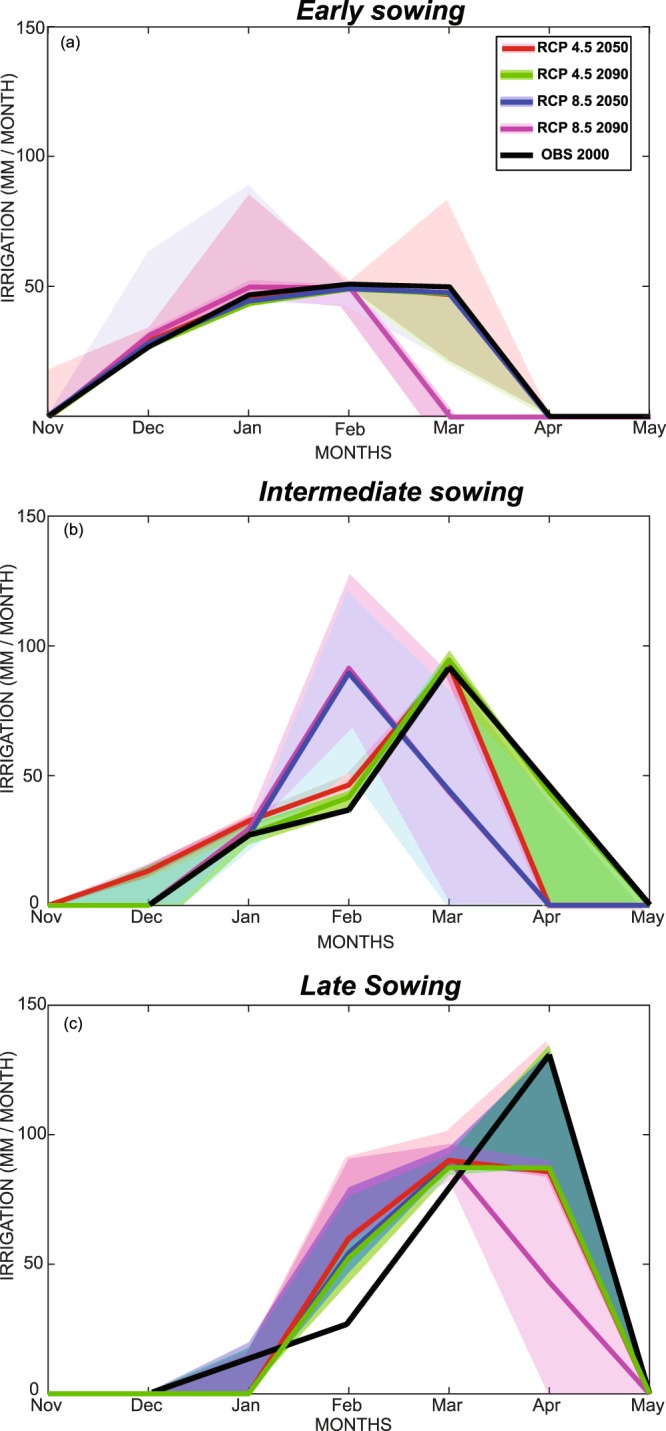
Irrigation for (**a**) early, (**b**) intermediate and (**c**) late sowing (“CCO2” experiment). The line is the model mean and the colored section correspond to Mean ± SD of the 5 RCM models.

Figure 4 displays irrigation requirements (IR) only, for the three sowing date and for all scenarios and horizons. For early sowing, current IRs are about 160 mm distributed from December to March with a maximum in February (48 mm). In 2050 according to the RCP4.5 scenario, IR requirements should increase by 2% to 164 mm, again with a peak in February. By contrast in 2090, according to RCP8.5, IR should decrease of 33 mm or 20% from December to February, with the maximum advanced by one month to January (49 mm). For late and intermediate sowing, the projections of IR follow the same decreasing trend in irrigation. The shift in peak requirement is about 2 months for late sowing in the more extreme scenario and for the later horizon. Decreases in the maximum are due to decreases in the temperature and reference evapotranspiration in February as compared to April. Decrease of evapotranspiration is due both to a shorter growing season and to improved stomatal regulation.

#### Impact of CC on water productivity (WP)

Water productivity (WP) is expressed as the dry matter above ground (gr or kg) produced per unit of transpired water (mm). Since the fertilizing effect in AquaCrop consists of modulating the Water Productivity parameter, an increase in the efficiency of water use is obviously expected (Fig. S3). The results show that actual WPs are 2.1, 1.6 and 1.3 kg/m^3^ for early, intermediate and late sowing respectively. With a rise in CO_2_ concentration plus a temperature increase, WPs are expected to increase for all climate scenarios up to 93% for RCP8.5 at the 2090 horizon, depending on the sowing dates. Hunsaker *et al*.^54^ demonstrated that the water productivity of wheat could increase by 50 to 60% depending on the water conditions under a doubled CO_2_ concentration. This increase in WP is due to increased photosynthesis^55^ and thus increased grain production per unit of transpired water^56^.

## Discussion and Conclusion

A systematic increase in maximum and minimum temperature for all scenarios and horizons is observed. This temperature rise ranges from 1 °C (RCP4.5 by 2050) to 6 °C (RCP8.5 by 2090) for yearly averages. The changes in temperature and precipitation are in agreement with several studies signalling (1) a significant temperature rise for the entire Mediterranean region^2,57^ or for the Tensift catchment^58^ and (2) a decrease in annual precipitation in the northern Mediterranean ranging from 16% in 2036–2065 for the RCP4.5 scenario to 30% in 2066–2095 for RCP8.5^21^. Likewise, the associated increase of the reference evapotranspiration ET0 was also highlighted in line with the results of Saadi *et al*.^17^ and Tramblay *et al*.^21^. The seasonality of the changes points out several implications for wheat growth and production. The decrease in precipitation is particularly prominent in spring (March and April) during the grain filling stage and autumn (October to December) around emergence. Early-sowing wheat could be particularly impacted by the temperature rise because of a greater warming in winter than in autumn and spring. The decrease of cycle duration could reach 32% (50 days) in 2090 according to scenario RCP8. 5. This value is closed to the work of Saadi *et al*.^17^ and Wang *et al*.^44^ who have previously shown that temperature rise predicted in Mediterranean regions could induce a reduction of the LCS ranging from 22 to 41 days as a consequence of 2.3–3.8 °C increases in air temperature.

Higher atmospheric CO_2_ concentrations increased yield for all climate scenarios and horizons. This strongly positive effect was expected as CO_2_ fertilization has been proven to be maximized on warm and dry environments^59^. Similar trends have already been reported from modeling studies by Ludwig and Asseng^15^ and Wang *et al*.^60^ who have shown that doubling atmospheric CO_2_ could increase yields by up to 48% using the APSIM-wheat. Interestingly, increases of the same order of magnitude have been obtained from experimental studies such as Amthor^61^, who obtained almost 31% higher grain yields by doubling the CO_2_ concentration value under laboratory conditions or Fitzgerald *et al*.^62^ who measured a CO_2_ stimulation of wheat production ranging from 24% to 53% in FACE experiments. By contrast, temperature rise at constant atmospheric CO_2_ concentration result in lower yields for all climate scenarios and sowing dates. In line with our results, Ludwig and Asseng^15^ found that a rise of 4 °C decreased the potential yield by as much as 32% and that an increase of 6 °C decreased the yield a further 50%. Also, You *et al*.^63^ observed a significant reduction in yield in China owing to a rise in temperature and it was concluded that a 1.8 °C rise could cause a 3–10% reduction in wheat yields. Early sowing could be significantly more heavily impacted by temperature rise. When considering climate change and rising CO_2_ concentration, it appears that whatever the scenario and the horizon, the fertilizing effect of atmospheric CO_2_ concentration might offset the losses induced by rising temperatures. Interestingly, the resulting increase of yields is higher for intermediate and late sowing. The simulated positive interaction between elevated CO_2_ concentration and temperature was also observed in several experimental studies^61^. Wheeler *et al*.^64^ have grown winter wheat (*Triticum aestivum*) in a field inside polyethylene-covered tunnels at a range of temperatures from about 1–2 °C below to about 2–3 °C above ambient, and at CO_2_ concentrations of 380 and 684 ppm. They found that grain yield was reduced by warmer temperatures but increased by CO_2_ enrichment at all temperatures. The grain-filling rate could also be impacted by the combined effects of temperature and CO_2_ increase. Wheeler *et al*.^64^ again found that the rate of increase in the dry weight of grain per ear was 8.0 mg/day greater at 684 ppm than at 380 ppm CO_2_ concentration for a given temperature, but that this higher filling rate was not able to compensate for the reduction of the grain-filling rate caused by increased temperature.

Water requirements decrease for all scenarios and horizons taking into account temperature and precipitation change and the CO_2_ fertilizing effect. This results from a drop in both cumulative evaporation and transpiration because of the shortening of the wheat cycle and improved stomatal closure respectively. Atkinson *et al*.^65^ and Hendrey *et al*.^66^ observed a decrease of stomatal conductance of 30%-40% although there are large differences between species. Woodward^67^ has also highlighted a decline in stomatal density and concluded from these changes that the efficiency of water use has improved significantly. Hunsaker *et al*.^54^ demonstrated that the water productivity of wheat could increase by 50 to 60% depending on the water conditions under a doubled CO_2_ concentration. This increase in WP is due to increased photosynthesis^55^ and thus increased grain production per unit of transpired water^56^. The irrigation requirement decreases by 11 to 31% depending on the scenario and horizon. Interestingly, the decrease in the cycle’s duration could also impact the temporal pattern of irrigation, with a possible shift of the season towards a requirement peak as much as two months earlier for the most extreme case.

Despite a number of studies^39,68^ showing that rising CO_2_ concentration negates the adverse effects of rising temperatures in semi-arid climates, it is important to reemphasize that the uncertainties remain high. In fact, the sign of change is reversed whether or not the rise in CO_2_ concentration is taken into account. “Reality” will certainly lie in between. The first issue to be adressed^69^ is the parameterization of detailed, process-based photosynthetic models such as that of Farquhar *et al*.^70^ for C3 plants. This might better represent the complex interaction between CO_2_ and the meteorological environment in plant physiology than semi-empirical approaches such as AquaCrop’s. In addition, several factors that have not been considered in this study. Another important aspect is related to the soil type, which may alter wheat production via the soil’s holding capacity. Larger negative impacts from rising temperatures are usually expected on light soils with lower water-holding capacities^15,16^. This means in particular that wheat in the Tensift region seems to bring together a number of favorable conditions favoring yield increase in a changing climate (heavy soils, a warm and dry climate favoring a positive reaction to CO_2_ rise).

The results could have direct implication for agricultural practices and water management in the region. First, considering water requirement, the seasonal pattern of irrigation demand could be significantly modified, with a peak requirement that might be advanced for wheat by about two months. This change in the temporal pattern of irrigation requirement could affect water managers, who could reap more benefit from surface water for irrigating cereals in winter while spring inputs, partially originating from snow melt in snowfed catchments such as the Tensift, could be retained to water perennials during the summer. This point is important within the context of the current extension and intensification of tree crops in the Mediterranean area, which further constrain agricultural water demand, especially during the hottest months^71,72^. The precipitation decrease in autumn could also foster a later sowing and shift the season by up to two months as farmers usually seed after the first rainfall, even for irrigated areas. While early sowing has been found to have a positive impact on final production for Mediterranean areas^73^ by avoiding coincidence between the pollination and period of high temperature^14^, our study suggest that in a changing climate later sowing could be preferable. Another question that arises from the shortening of the growth cycle is related to the optimal choice of wheat cultivars. Long-duration wheat varieties are not well suited to the semi-arid Mediterranean region at the present time because the grain-fillling phase coincides with the severe temperatures occuring in late spring. In view of the shortening of the crop cycle, longer duration and drought-tolerant varieties that may prove more productive^74^ could become increasingly rewarding in a changing climate.

Finally, we evaluated only the impact of climate change on the optimal wheat yields and crop water needs without considering constraints in terms of water availability, agricultural practices and socio-economic conditions. In practice, the potential yield increase under climate change shown in this study under optimal conditions in terms of irrigation and fertilizer could never been reached because of several practical constraints. First, nitrogen is a major limiting factor for agricultural production. FACE experiments conducted on wheat by Long *et al*.^26^ with N inputs of 15 to 70 kg ha^−1^, which is considered to be low by all agricultural standards but exceeds the typical farm inputs in the study region, resulted in a yield increase of 9% only, about a third of what was expected^26^. To reach the simulated increase in yield, a nitrogen amount may be needed, well beyond the financial capacity of most small-scale farmers cropping wheat in the region. Another main constraint will be the availability of water resource in the future, due to reduced rainfall and increased evaporation demand. In a recent study, Tramblay *et al*.^21^ highlighted a potential decrease of surface water availability in Morocco (dams are the principal source of irrigation) that could reach 40% around 2066–2095 under RCP8.5 scenario. Within this context, water allocated to irrigation could decrease in the future considering the numerous competing demands (industry, tourism, drinking water …). In reality, irrigation efficiency is also far from optimal. Taking into account leakage within the network, bad irrigation scheduling and water losses by drainage and soil evaporation, irrigation efficiency barely exceed 50%^75^. Considering only irrigation technics and farmers practices, several studies carried out in Morocco^76–79^ based on experimental data (mainly eddy-covariance stations, lysimeters and soil moisture profile) showed very different results in terms of water lost by the plant either through soil evaporation or deep drainage. The main conclusion is that the conversion to drip irrigation that is fostered in several north african countries through ambitious public policy does not necessary lead to the expected water savings with regards to more traditional method such as flooding. The reason are numerous: bad use of the technic by the farmers, soil washout because of a high level of salinity, extension of irrigation to the surrounding field that were not previously irrigated because of an easier access to water thanks to the drip system, intensification of crops (inter-cropping) for the same reason … By contrast, irrigation scheduling could also be modified towards deficit irrigation consisting in a trade-off between water saving while maintaining acceptable level of yields in order to save water.

This study points out the need (1) for more field experiments in order to better understand how temperature, water and CO_2_ concentration interact to impact wheat yields; (2) for considering agricultural practices in future studies.

## Methods

### Study site and meteorological data

**Figure 5:**
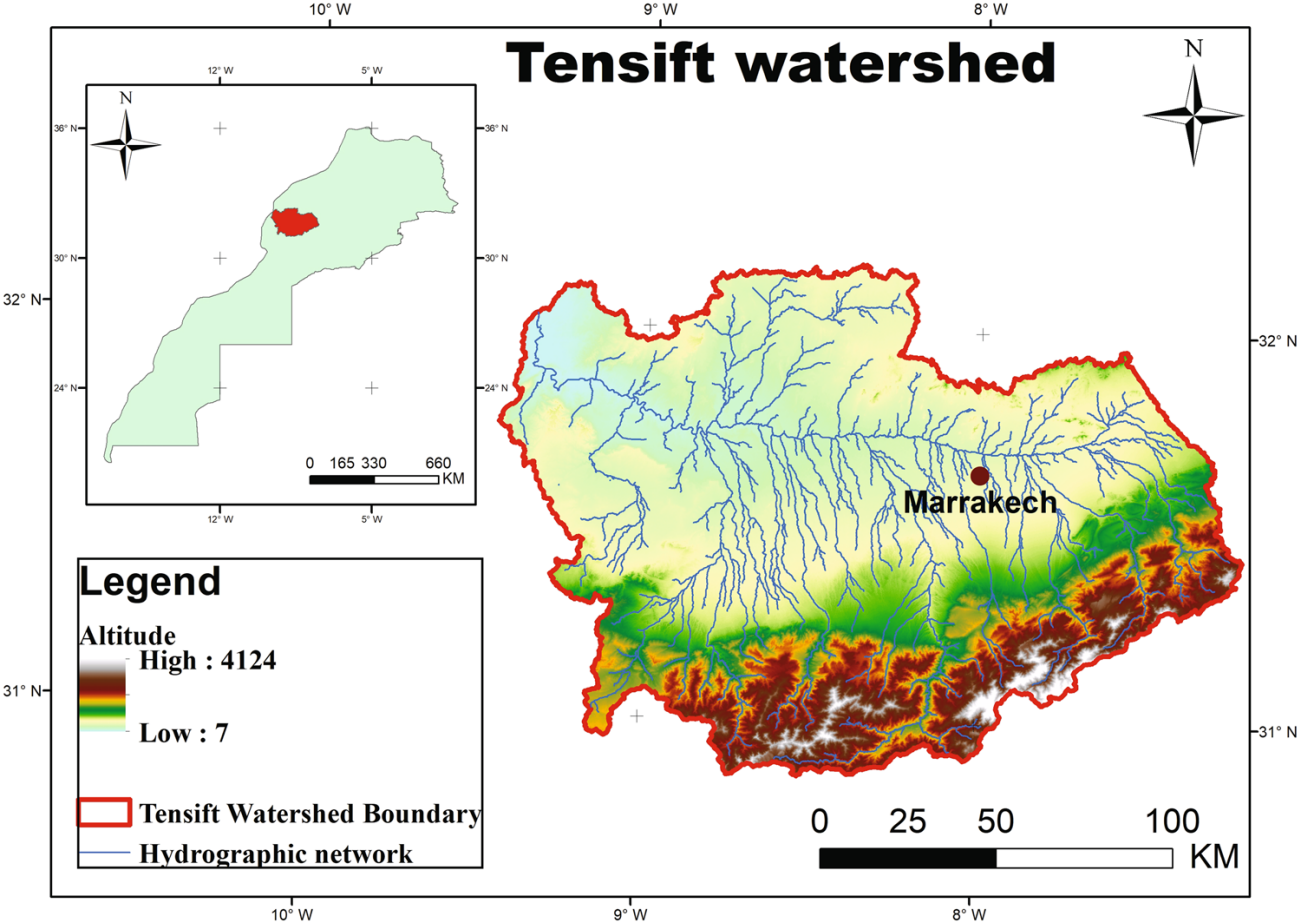
Location of the Tensift watershed.

The Tensift-Haouz region, located in the center of Morocco (Fig. 5), covers some 20,000 km² and is characterized by a semi-arid Mediterranean climate. The atmosphere is dry, with an average relative humidity of 56%^15,47,48^. The annual average evaporation demand is very high (around 1600 mm/year), based on the reference evapotranspiration $E{T}_{0}$^80^, greatly exceeding the annual rainfall which ranges from 190 to 250 mm/year^81^. Most of the precipitation falls during winter and spring from the beginning of November until the end of April^15,47,48^. Common wheat is one of the region’s main crops. It is cultivated both in rainfed and irriga ted fields, depending on access to water supply and climate conditions. Cereals can be sown as early as November 1^st^ if significant rainfall occurs, but a persistent drought at the beginning of the growing season can delay seeding until January 15^th^. Harvesting usually takes place around the end of May. Precipitation, minimum and maximum temperature data were acquired by the synoptic station at Marrakech airport located at 8°W/31°30’N from 1991 to 2010.

### Climate data and bias corrected scenarios

Five regional climate model (RCM) simulations with a horizontal resolution of 50 km were extracted from the Med-CORDEX experiment^82^, which aims to produce a set of simulations at various resolutions, taking into account the specificity of the Mediterranean climate. The main characteristics of each simulation are presented in Table S1.

Two climate scenarios were selected for comparison: the representative concentration pathways (RCP) 4.5 and 8.5. Emissions in the RCP4.5 peak around 2040, then decline, while in RCP8.5, emissions continue to rise throughout the 21st century. These RCPs scenarios and their associated atmospheric CO_2_ concentrations are listed in Table S2. Finally, two time horizons are considered (2041–2060 and 2081–2100) and compared to the so-called “historical” period (1991–2010).

For each model, the following climate simulations were selected:

- Historical runs (HIST): simulations for the control period available from 1950‒2005 depending on the model, with RCMs forced by the various GCMs indicated in Table S1.
- Scenario runs: simulations for future projections (2005‒2100) with RCMs forced by various GCMs according to emission scenarios RCP4.5 and RCP8.5.

Extracted variables correspond to the meteorological inputs of the AquaCrop model at a daily time step: minimum temperature (Tmin), maximum temperature (Tmax) and precipitation (pr). Reference evapotranspiration was estimated using the empirical method Hargreaves–Samani which requires only air temperature^83^. Er-Raki *et al*.^81^ have shown the good performance of this formula in Tensift region. Although the RCM simulations have been found to match the main patterns of climates encountered in Mediterranean countries^84,85^, some significant and systematic discrepancies with observed data have been highlighted. For this reason, bias correction of climate runs is unavoidable for hydrological and agronomical impact studies. The five Med-CORDEX runs corresponding to the grid point centered on the synoptic station at Marrakech (8°W/31°30’N) are thus bias corrected based on the daily data acquired by the station using the quantile-quantile approach described below. Only temperature and precipitation are bias corrected in this study.

The bias correction method aims to correct the large discrepancies usually observed between model predictions and local observations that are attributed to parameterization problem or to orography representation. The quantile-quantile method has the advantage of correcting skews of the considered model. It is a non-linear method which consists in correcting the values of the quantiles of the model by those calculated from the observations^86^. In each point of the model, for each weather variable, the 99 percentiles of the daily series are then calculated as well as the minimum and maximum values and the same is done from the series of daily observations. The correction function consists of associating each percentile of the model with the observed percentile, and, thus, of matching the distribution of observations to the simulations. For any model value between two percentiles, a linear interpolation is carried out^86^. The software used in this study has been developed by Giulani *et al*.^87^. Within this study, the bias correction of daily temperature and precipitation has been applied to all models separately in order to assess the ensemble spread. In addition, it was independently applied season by season (January-February-March, April-May-June, July-August-September, October-November-December). This was done because of the strong seasonal contrast of rainfall distribution between the rainy season (from November to May) and the dry season during the summer. In order to assess the impact of the climate scenarios spread on yields and water requirements projections, three simulations of the Aquacrop model were carried out using the Mean and Mean ± SD of the 5 RCM models.

A literature review suggests that bias correction methods including the quantile-quantile used in this study suffer from different caveats^88,89^: (1) it does not always preserve trends of the raw GCM runs^90^; (2) it tends to overestimate the spread of the ensemble runs of climate models for future projected variables^91^; (3) it can also corrupts the extremes (lower and higher percentiles)^92,93^. As these limitations could have some implications for the conclusion of our study, it has been checked that the trends of the raw climate runs together with the spread of the ensemble is not significantly modified by the application of the quantile-quantile approach (not shown). Finally, the corruption of very extreme lower and higher percentiles is a common feature of bias correction method. For our study, it precludes from studying the impact of threshold processes such as during the pollination stage when very high temperature (above 30 °C for daily average) lead to a drastic drop of wheat (cf. description of the Aquacrop model in the supplementary material).

### The Aquacrop model

AquaCrop, developed by the Land and Water Division of the Food and Agriculture Organization of the United Nations (FAO), is a crop model to simulate yield response to water use as a decision support tool for agricultural planning and scenario analysis including future climate scenario^30,94,95^. AquaCrop includes the following sub-model components: soil, crop, atmosphere and management^96^. The impact of climate change can be evaluated in AquaCrop by: (i) adjusting the precipitation data, (ii) adjusting the temperature data, (iii) enhancing the CO_2_ levels. The first two options are performed in this study through the Med-CORDEX ensemble runs described above. In addition, the CO_2_ rise scenarios from RCP4.5 and RCP4.8 were applied (Table S2). The basic concepts and fundamental calculation procedures of AquaCrop are presented in Steduto *et al*.^30^ and further described as supplementary materials (section S1).

### Implementation of AquaCrop and experiments

The FAO offers calibrated crop parameter values for most agricultural crops and provides them as default values in the model. In particular, a distinction is made between conservative, and non-conservative parameter:

- Conservative crop parameters are not materially affected by time, management practices or geographic location (for example: base temperature, upper temperature and initial canopy cover). They were calibrated using data from crops grown under favorable and non-limiting conditions^30^.
- Non-conservative crop parameters (Calibrated parameter) may require adjustment when selecting a different variety from that used for calibration (for example: time from sowing to emergence, maximum canopy cover, start of senescence). Non-conservative crop parameters are influenced by plot management, soil profile conditions, and climate.

In our study, the generic variety of wheat is *Triticum durum* and the sowing density is 150 kg/ha. Both parameters were kept identical for each experiment (see below). The soil is homogeneous, characterized by a clay-loamy texture typical of the region of study: the values of field capacity (Fc), permanent wilting point (PWP), saturation (Sat) and hydraulic conductivity (Ksat) were 0.32, 0.17, 0.45 m^3^/m^3^ and 100 mm/day respectively. In addition, non-conservative calibrated parameters for winter wheat crop in the Tensift region were obtained from Toumi *et al*.^31^ (Table S3). Various parameters affecting canopy cover (FC), evapotranspiration, total water content (TWC) and yield were calibrated and validated on the basis of a comparison between measurements and the results of simulations^31^. The average values of the Mean Bias Error (MBE) between observed and measured CC, evapotranspiration and GY were 7.89%, −0.01 mm/day and 0.06 t/ha for the validation fields, respectively. Finally, since the focus is on irrigated wheat, irrigation is planned automatically by the model to avoid water stress. The AquaCrop model calculates the quantity of water required to avoid water stress on the crop. When the depletion of the root zone exceeds a certain threshold (Dr = 0.5 TAW in our case following Toumi *et al*.^31^), an automatic irrigation is carried out to reset the soil depletion. This calibrated version of the Aquacrop model is thus assumed to simulate with a reasonable accuracy yields and water requirements for wheat in our region of study.

Three typical sowing dates were evaluated: early sowing around November 15^th^; intermediate sowing around December 15^th^; and late sowing around January 15^th^. In order to assess the actual optimal wheat yields and water requirements, AquaCrop was run with observational data (“historical” experiment) with a climatology forcing corresponding to 20 years (1991–2010) using CO_2_ concentrations, with the value of year 2000 set to 369 ppm. The results of this experiment were then used to determine the baseline of wheat yield, water requirements and productivity, from which any increases or decreases due to climate change could be estimated. In future-climate scenarios, the effects of increasing CO_2_ concentration were isolated from predicted changes in climate variables (precipitation and temperature) with the help of three separate experiments: a CO_2_-only change named the “CO_2_” experiment; climate change only, including temperature and precipitation changes (“CC” experiment); and CO_2_ and climate changes (“CCO2” experiment). For experiments taking into account a change in CO_2_ concentration (“CO_2_” and “CCO2”), the ambient atmospheric CO_2_ was replaced by the values from the two RCP scenarios (Table S2). For each scenario, projections are carried out under two scenarios (RCP4.5, RCP8.5) and two time horizons (2041–2060, 2081–2100). For the “CC” and “CCO2” experiments, AquaCrop was run with average of climate data and average plus and minus standard deviations between all RCM models to assess the uncertainties of projections associated with climate ensemble runs.

**Figure 6:**
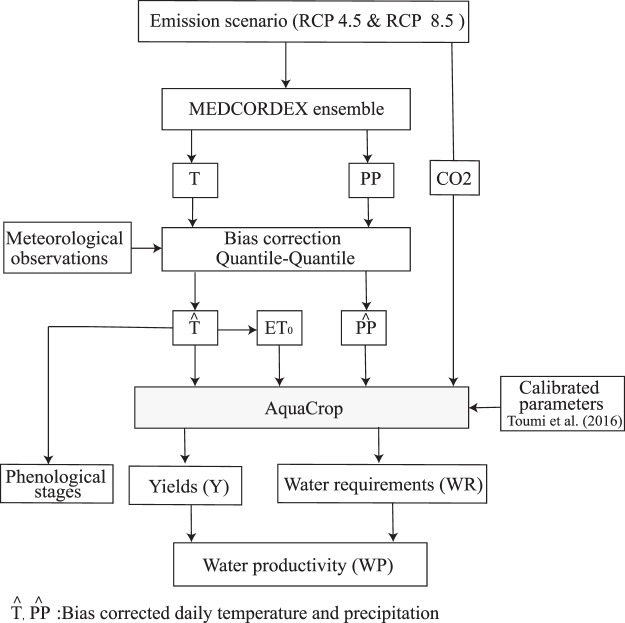
Flowchart of the proposed methodology to study the impact of climate change on grain yield (Y), water requirements (WR) and water productivity (WP).

A summary of the data analysis and simulation steps used in this study is presented in the flowchart below (Fig. 6).

## Supplementary information

Supplementary Information

## References

[1] IPCC. IPCC Fifth Assessment Synthesis Report-Climate Change 2014 Synthesis Report. *I**PCC Fifth Assess. Synth. Report-Climate Chang. 2014 Synth. Rep*. pages: 167 (2014).

[2] Giorgi F (2006). Climate change hot-spots. Geophys. Res. Lett..

[3] Chiffres, L. M. en. Sommaire Préambule. **537**, 212–66 (2015).

[4] Blinda, M. & Giraud, J. Vers une meilleure efficience de l’utilisation de l’eau en Méditerranée. *LES CAHIERS DU PLAN BLEU N°14* **44** (2012).

[5] Lobell DB (2008). Prioritizing climate change adaptation needs for food security in 2030. Science (80-.)..

[6] Morison, J. I. L. Intercellular CO_2_ concentration and stomatal response to CO_2_. in *Stomatal function* 229–252 (1987).

[7] Drake BG, Gonzàlez-Meler MA, Long SP (1997). MORE EFFICIENT PLANTS: A Consequence of Rising Atmospheric CO_2_?. Annu. Rev. Plant Physiol. Plant Mol. Biol..

[8] Kimball, B. A. *et al*. Elevated CO_2_, drought and soil nitrogen effects on wheat grain quality. *New Phytol*. **150** (2001).

[9] Herwaarden AF, van, Farquhar GD, Angus JF, Richards RA, Howe GN (1998). ‘Haying-off’, the negative grain yield response of dryland wheat to nitrogen fertiliser. I. Biomass, grain yield, and water use. Aust. J. Agric. Res..

[10] Burke JJ, Mahan JR, Hatfield JL (1988). Crop-Specific Thermal Kinetic Windows in Relation to Wheat and Cotton Biomass Production. Agron. J..

[11] Mearns LO, Rosenzweig C, Goldberg R (1997). Mean and Variance Change in Climate Scenarious: Methods, Agricultural Applications and Measures of Uncertainity. Clim. Change.

[12] Peng S (2004). Rice yields decline with higher night temperature from global warming. Proc. Natl. Acad. Sci..

[13] Hafid, R. E, Smith, D. H., Karrou, M. & Samir, K. Morphological attributes associated with early-season drought tolerance in spring durum wheat in a mediterranean environment. *Crop Sci*. 273–282 (1998).

[14] Heng LK, Asseng S, Mejahed K, Rusan M (2007). Optimizing wheat productivity in two rain-fed environments of the West Asia–North Africa region using a simulation model. Eur. J. Agron..

[15] Ludwig F, Asseng S (2006). Climate change impacts on wheat production in a Mediterranean environment in Western Australia. Agric. Syst..

[16] Yang Y, Liu DL, Anwar MR, Zuo H, Yang Y (2014). Impact of future climate change on wheat production in relation to plant-available water capacity in a semiaridenvironment. Theor. Appl. Climatol..

[17] Saadi S (2015). Climate change and Mediterranean agriculture: Impacts on winter wheat and tomato crop evapotranspiration, irrigation requirements and yield. Agric. Water Manag..

[18] Ruiz-Ramos M, Mínguez MI (2010). Evaluating uncertainty in climate change impacts on crop productivity in the Iberian Peninsula. Clim. Res..

[19] Wang B, Liu DL, Asseng S, Macadam I, Yu Q (2017). Modelling wheat yield change under CO_2_ increase, heat and water stress in relation to plant available water capacity in eastern Australia. Eur. J. Agron..

[20] Lovelli S (2010). Effects of rising atmospheric CO_2_ on crop evapotranspiration in a Mediterranean area. Agric. Water Manag..

[21] Tramblay Y, Jarlan L, Hanich L, Somot S (2018). Future Scenarios of Surface Water Resources Availability in North African Dams. Water Resour. Manag..

[22] Dettori M, Cesaraccio C, Duce P (2017). Simulation of climate change impacts on production and phenology of durum wheat in Mediterranean environments using CERES-Wheat model. F. Crop. Res..

[23] Valverde P (2015). Climate change impacts on rainfed agriculture in the Guadiana river basin (Portugal). Agric. Water Manag..

[24] Rodriguez-Diaz AJ, Weatherhead EK, Knox JW, Camacho E (2007). Climate change impacts on irrigation water requirements in the Guadalquivir river basin in Spain. Reg. Environ. Chang..

[25] Fader M, Shi S, Von Bloh W, Bondeau A, Cramer W (2016). Mediterranean irrigation under climate change: More efficient irrigation needed to compensate for increases in irrigation water requirements. Hydrol. Earth Syst. Sci..

[26] Long SP, Ainsworth EA, Leakey ADB, Ort DR, No J (2006). Food for Thought: Lower-Than-Expected Crop Yield Stimulation with Rising CO_2_ Concentrations. Science (80-.)..

[27] Tubiello FN, Ewert F (2002). Simulating the effects of elevated CO_2_ on crops: approaches and applications for climate change. Eur. J. Agron..

[28] Ainsworth EA, Long SP (2005). What have we learned from 15 years of free-air CO_2_ enrichment (FACE)? A meta-analytic review of the responses of photosynthesis, canopy properties and plant production to rising CO_2_. New Phytol..

[29] Yin X (2013). Improving ecophysiological simulation models to predict the impact of elevated atmospheric CO_2_ concentration on crop productivity. Ann. Bot..

[30] Steduto P, Hsiao TC, Raes D, Fereres E (2009). AquaCrop—The FAO Crop Model to Simulate Yield Response to Water: I. Concepts and Underlying Principles. Agron. J..

[31] Toumi J (2016). Performance assessment of AquaCrop model for estimating evapotranspiration, soil water content and grain yield of winter wheat in Tensift Al Haouz (Morocco): Application to irrigation management. Agric. Water Manag..

[32] Akumaga U, Tarhule A, Piani C, Traore B, Yusuf A (2018). Utilizing Process-Based Modeling to Assess the Impact of Climate Change on Crop Yields and Adaptation Options in the Niger River Basin, West Africa. Agronomy.

[33] Bird DN (2016). Modelling climate change impacts on and adaptation strategies for agriculture in Sardinia and Tunisia using AquaCrop and value-at-risk. Sci. Total Environ..

[34] Abedinpour M, Sarangi A, Rajput TBS, Singh M (2014). Prediction of maize yield under future water availability scenarios using the AquaCrop model. J. Agric. Sci..

[35] Stevens T, Madani K (2016). Future climate impacts on maize farming and food security in Malawi. Sci. Rep..

[36] Vanuytrecht E, Raes D, Willems P (2011). Considering sink strength to model crop production under elevated atmospheric CO_2_. Agric. For. Meteorol..

[37] Vanuytrecht E, Raes D, Willems P, Geerts S (2012). Quantifying field-scale effects of elevated carbon dioxide concentration on crops. Climate Research.

[38] McMaster GS, Wilhelm WW (1997). Growing degree-days: One equation, two interpretations. Agric. For. Meteorol..

[39] Dixit PN, Telleria R, Al Khatib AN, Allouzi SF (2018). Decadal analysis of impact of future climate on wheat production in dry Mediterranean environment: A case of Jordan. Sci. Total Environ..

[40] Ewert F (2002). Effects of elevated CO_2_ and drought on wheat: Testing crop simulation models for different experimental and climatic conditions. Agric. Ecosyst. Environ..

[41] Pinter PJ (2000). Free-air CO_2_ enrichment (FACE): Blower effects on wheat canopy microclimate and plant development. Agric. For. Meteorol..

[42] Lawlor, D. W. & Mitchell, R. A. C. Crop ecosystem responses to climatic change: wheat. *Clim. Chang. Glob. Crop Product*. 57–80, 10.1079/9780851994390.0057 (2000).

[43] Jarlan, L. *et al*. Linkages between common wheat yields and climate in Morocco (1982-2008). *Int. J. Biometeorol*. **58** (2014). 10.1007/s00484-013-0753-9 24177944

[44] Wang J, Wang E, Liu DL (2011). Modelling the impacts of climate change on wheat yield and field water balance over the Murray-Darling Basin in Australia. Theor. Appl. Climatol..

[45] Wardlaw IF, Dunstone RL (1984). Effect of temperature on seed development in jojoba (Simmondsia chinensis (Link) Schneider). I. Dry matter changes. Aust. J. Agric. Res..

[46] Hatfield JL, Prueger JH (2015). Temperature extremes: Effect on plant growth and development. Weather Clim. Extrem..

[47] Duchemin B (2006). Monitoring wheat phenology and irrigation in Central Morocco: On the use of relationships between evapotranspiration, crops coefficients, leaf area index and remotely-sensed vegetation indices. Agric. Water Manag..

[48] Er-Raki S (2007). Combining FAO-56 model and ground-based remote sensing to estimate water consumptions of wheat crops in a semi-arid region. Agric. Water Manag..

[49] Aouade G., Ezzahar J., Amenzou N., Er-Raki S., Benkaddour A., Khabba S., Jarlan L. (2016). Combining stable isotopes, Eddy Covariance system and meteorological measurements for partitioning evapotranspiration, of winter wheat, into soil evaporation and plant transpiration in a semi-arid region. Agricultural Water Management.

[50] Diarra A (2017). Performance of the two-source energy budget (TSEB) model for the monitoring of evapotranspiration over irrigated annual crops in North. Africa. Agric. Water Manag..

[51] Rafi Z (2019). Partitioning evapotranspiration of a drip-irrigated wheat crop: Inter-comparing eddy covariance-, sap flow-, lysimeter- and FAO-based methods. Agric. For. Meteorol..

[52] Hadria, R. *et al*. Calibration and validation of the STICS crop model for managing wheat irrigation in the semi-arid Marrakech/Al Haouzi plain. *Arab. J. Sci. Eng*. (2007).

[53] Kharrou MH (2013). Assessment of Equity and Adequacy of Water Delivery in Irrigation Systems Using Remote Sensing-Based Indicators in Semi-Arid Region, Morocco. Water Resour. Manag..

[54] Hunsaker D (2000). CO2 enrichment and soil nitrogen effects on wheat evapotranspiration and water use efficiency. Agricultural and Forest Meteorology.

[55] Stoddard, F. L., Mäkelä, P. S. A. & Puhakainen, T. Adaptation of Boreal Field Crop Production to Climate Change. *Clim. Chang. - Res. Technol. Adapt. Mitig*. (2011).

[56] Fischer G, Tubiello FN, Velthuizen HV, Wiberg DA (2007). Climate change impacts on irrigation water requirements: Effects of mitigation, 1990–2080. Technol. Forecast. Soc. Change.

[57] Haim D, Shechter M, Berliner P (2008). Assessing the impact of climate change on representative field crops in Israeli agriculture: A case study of wheat and cotton. Clim. Change.

[58] Marchane A, Tramblay Y, Hanich L, Ruelland D, Jarlan L (2017). Climate change impacts on surface water resources in the Rheraya catchment (High Atlas, Morocco). Hydrol. Sci. J..

[59] Idso SB, Kimball BA, Mauney JR (1988). Atmospheric CO_2_ enrichment and plant dry matter content. Agric. For. Meteorol..

[60] Wang J, Wang E, Luo Q, Kirby M (2009). Modelling the sensitivity of wheat growth and water balance to climate change in Southeast Australia. Clim. Change.

[61] Amthor JS (2001). Effects of atmospheric CO_2_ concentration on wheat yield: Review of results from experiments using various approaches to control CO_2_ concentration. F. Crop. Res..

[62] Fitzgerald GJ (2016). Elevated atmospheric [CO_2_] can dramatically increase wheat yields in semi-arid environments and buffer against heat waves. Glob. Chang. Biol..

[63] You L, Rosegrant MW, Wood S, Sun D (2009). Impact of growing season temperature on wheat productivity in China. Agric. For. Meteorol..

[64] Wheeler TR (1996). The duration and rate of grain growth, and harvest index, of wheat (*Triticum aestivum* L.) in response to temperature and CO _2_. J. Exp. Bot..

[65] Atkinson CJ, Wookey PA, Mansfield TA (1991). Atmospheric pollution and the sensitivity of stomata on barley leaves to abscisic acid and carbon dioxide. New Phytol..

[66] Hendrey GR, Ellsworth DS, Lewin KF, Nagy J (1999). A free-air enrichment system for exposing tall forest vegetation to elevated atmospheric CO_2_. Glob. Chang. Biol..

[67] Woodward, F. I. Plant resonses to past concentrations of carbon dioxide. 145–155 (1993).

[68] Angulo C (2013). Implication of crop model calibration strategies for assessing regional impacts of climate change in. Europe. Agric. For. Meteorol..

[69] Soussana JF, Graux AI, Tubiello FN (2010). Improving the use of modelling for projections of climate change impacts on crops and pastures. J. Exp. Bot..

[70] Farquhar GD, von Caemmerer S, Berry JA (1980). A biochemical model of photosynthetic CO_2_ assimilation in leaves of C3 species. Planta.

[71] Jarlan, L. *et al*. Water Resources in South Mediterranean Catchments: Assessing climatic drivers and impacts. in *The Mediterranean Region under Climate Change* (eds. Thiébault, S. & Moatti, J. P.) 303–309 (2016).

[72] Voltz Marc, Ludwig Wolfgang, Leduc Christian, Bouarfa Sami (2018). Mediterranean land systems under global change: current state and future challenges. Regional Environmental Change.

[73] Photiades I, Hadjichristodoulou A (1984). Sowing date, sowing depth, seed rate and row spacing of wheat and barley under dryland conditions. F. Crop. Res..

[74] Boote KJ (2011). Position statement on crop adaptation to climate change. Crop Sci..

[75] Hamdy, A. & Katerji, N. Water crisis in the Arab World. Analysis and solutions. in *I**AM-Bari**Editor* 60 p. (2006).

[76] Khabba S (2013). The SudMed Program and the Joint International Laboratory TREMA: A Decade of Water Transfer Study in the Soil-plant-atmosphere System over Irrigated Crops in Semi-arid Area. Procedia Environ. Sci..

[77] Nassah H (2018). Evaluation and analysis of deep percolation losses of drip irrigated citrus crops under non-saline and saline conditions in a semi-arid area. Biosyst. Eng..

[78] Sefiani S., El Mandour A., Laftouhi N., Khalil N., Chehbouni A., Jarlan L., Hanich L., Khabba S., Kamal S., Markhi A., Nassah H. (2019). Evaluation of Groundwater Quality and Agricultural use Under a Semi‐arid Environment: Case of Agafay, Western Haouz, Morocco. Irrigation and Drainage.

[79] Jarlan L (2015). Remote Sensing of Water Resources in Semi-Arid Mediterranean Areas: the joint international laboratory TREMA. Int. J. Remote Sens..

[80] Testa G., Gresta F., Cosentino S.L. (2011). Dry matter and qualitative characteristics of alfalfa as affected by harvest times and soil water content. European Journal of Agronomy.

[81] Er-Raki S., Chehbouni A., Khabba S., Simonneaux V., Jarlan L., Ouldbba A., Rodriguez J.C., Allen R. (2010). Assessment of reference evapotranspiration methods in semi-arid regions: Can weather forecast data be used as alternate of ground meteorological parameters?. Journal of Arid Environments.

[82] Ruti PM (2016). Med-CORDEX initiative for Mediterranean climate studies. Bull. Am. Meteorol. Soc..

[83] Hargreaves GH, Samani ZA (1985). Reference crop evapotranspiration from temperatur. Trans. ASAE.

[84] Flaounas E (2013). Precipitation and temperature space-time variability and extremes in the Mediterranean region: Evaluation of dynamical and statistical downscaling methods. Clim. Dyn..

[85] Vaittinada Ayar P (2016). Intercomparison of statistical and dynamical downscaling models under the EURO- and MED-CORDEX initiative framework: present climate evaluations. Clim. Dyn..

[86] Déqué M (2007). Frequency of precipitation and temperature extremes over France in an anthropogenic scenario: Model results and statistical correction according to observed values. Glob. Planet. Change.

[87] Giulani, M., Li, Y., Anghileri, A., C. S. A. T. Plan: Introduction, https://github.com/mxgiuliani00/ClimateScenarioAnalysisToolbox(2015).

[88] Ehret U, Zehe E, Wulfmeyer V, Warrach-Sagi K, Liebert J (2012). HESS Opinions ‘should we apply bias correction to global and regional climate model data?’. Hydrology and Earth System Sciences.

[89] Maraun Douglas (2016). Bias Correcting Climate Change Simulations - a Critical Review. Current Climate Change Reports.

[90] Grillakis MG, Koutroulis AG, Daliakopoulos IN, Tsanis IK (2017). A method to preserve trends in quantile mapping bias correction of climate modeled temperature. Earth Syst. Dyn..

[91] Chen J (2019). Bias correcting climate model multi-member ensembles to assess climate change impacts on hydrology. Clim. Change.

[92] Ali, H., Modi, P. & Mishra, V. Increased flood risk in Indian sub-continent under the warming climate. *Weather Clim. Extrem*. **25** (2019).

[93] Cannon AJ, Sobie SR, Murdock TQ (2015). Bias correction of GCM precipitation by quantile mapping: How well do methods preserve changes in quantiles and extremes?. J. Clim..

[94] Hsiao TC (2009). Aquacrop-The FAO crop model to simulate yield response to water: III. Parameterization and testing for maize. Agron. J..

[95] Steduto, P. *et al*. Performance review of AquaCrop - The FAO crop-water productivity model. *ICID 21st Int. Congr. Irrig. Drain*. 231–248 (2011).

[96] Araya A, Habtu S, Hadgu KM, Kebede A, Dejene T (2010). Test of AquaCrop model in simulating biomass and yield of water deficient and irrigated barley (Hordeum vulgare). Agric. Water Manag..

