# Supplementary material for: Assessing the impact of global climate changes on irrigated wheat yields and water requirements in a semi-arid environment of Morocco
Source: Sci Rep. 2019 Dec 16;9:19142. doi: 10.1038/s41598-019-55251-2 (PMC6915735; doi:10.1038/s41598-019-55251-2)
Supplement: Supplementary file 1 — Supplementary Information [file 41598_2019_55251_MOESM1_ESM.doc]

**Assessing the impact of global climate changes on irrigated wheat yields and water requirements in a semi-arid environment of Morocco**

**Bouras E.^1,2,3^, Jarlan L.^2,3*^, Khabba S.^3,4,5^, Er-Raki S.^1,3,4^, Dezetter A.^3,6^, Sghir F.^3,7^, Tramblay Y.^3,6^**

^1^LP2M2E, Département de Physique Appliquée, Faculté des Sciences et Techniques, Université Cadi Ayyad, (UCAM) Marrakech, Morocco

Emails: [elhoussaine.bouras@ced.uca.ma](mailto:elhoussaine.bouras@ced.uca.ma) , [bouras.elhoussaine@gmail](mailto:bouras.elhoussaine@gmail).com & [s.erraki@uca.ma](mailto:s.erraki@uca.ma)

^2^Centre d’Etudes Spatiales de la BIOsphère (CESBIO), Institut de Recherche pour le Développement (IRD), Toulouse, France

Email : [lionel.jarlan@cesbio.cnes.fr](mailto:lionel.jarlan@cesbio.cnes.fr)

^3^Joint International Laboratory TREMA, Université Cadi Ayyad (UCAM), Marrakech, Morocco

Email : [khabba@uca.ac.ma](mailto:khabba@uca.ac.ma)

**^4^**Center for Remote Sensing Applications (CRSA), University Mohammed VI Polytechnic (UM6P), Benguerir, Morocco

^5^LMME, Faculté des Sciences Semalia (FSS), Université Cadi Ayyad (UCAM), Marrakech, Morocco

^6^Laboratoire HydroSciences Montpellier (HSM), France

Emails : [yves.tramblay@ird.fr](mailto:yves.tramblay@ird.fr) [&Alain.Dezetter@umontpellier.fr](mailto:%26Alain.Dezetter@umontpellier.fr)

^7^Office Régional de Mise en Valeur Agricole du Haouz (ORMVAH),Marrakech, Morocco

Email : [sgridber@gmail.com](mailto:sgridber@gmail.com)

* **Corresponding author**: Dr. Lionel Jarlan

# Supplementary Methods

## Description of the Aquacrop model

The complexity of crop responses to water deficits leads to the use of empirical production functions as the most practical option for assessing crop yield response. Among the empirical function approaches available, the FAO Irrigation & Drainage equation expresses the yield response as a function of the crop water status (Eq.1).


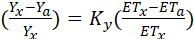
**Eq. 1**

where
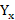
 and
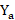
 are the maximum and actual yields,
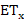
and
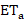
 are the maximum and actual evapotranspirations, and
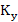
 is the proportionality factor between relative yield loss and relative reduction in evapotranspiration. The AquaCrop model uses canopy cover (FC) as the basis for calculating plant transpiration (
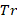
) and soil evaporation (
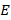
)^1^. Crop transpiration (
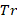
) is calculated by multiplying
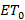
by the crop transpiration coefficient (
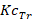
) and by considering the effect of water stress
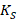
 (Eq.2).


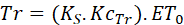
 **Eq. 2**


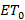
 is estimated with the Hargreaves equation^2^. Soil evaporation is calculated by multiplying
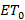
 by the soil water evaporation coefficient (
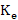
) and by considering the effect of soil water evaporation reduction
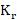
 (Eq.3):


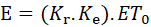
 **Eq. 3**

The separation of evapotranspiration into
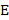
 and
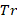
 avoids the confounding effect of the non-productive water^3^. Aboveground biomass is calculated as the product of the normalized crop water productivity (
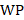
) and the accumulation of crop transpiration (
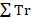
) as follows (Eq.4):


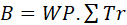
**Eq. 4**

where
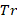
 is the crop transpiration (in mm/day) and
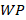
 is the water productivity parameter (kg of biomass per m^2^ and per mm of cumulated water transpired over the time period during which the biomass is produced).
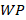
 is considered constant for a given climate and crop: it is set between 15 and 20 g m^-2^ for C3 crops such as wheat and between 30 and 35 g m^-2^ for C4 crops ^4^. The
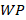
 parameter in the model is normalized in order to make the model applicable to diverse locations and seasons, including future climate scenarios^5,6^.

Crop yield
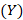
 is calculated as the product of biomass (
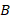
) and harvest index (
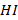
) (Eq. 5).


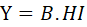
 ***Eq. 5***

The impact of increasing CO_2_ levels is calculated in AquaCrop by adjusting
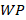
 using a correction coefficient
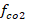
if the atmospheric CO_2_ concentration differs from its reference value (369 ppm) as follows:


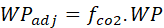
 **Eq. 6**

where
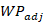
 is the
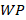
 adjusted for CO_2_ concentration and
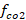
 represents the difference between the reference value and the atmospheric composition for a specific year, as follows ^4^:


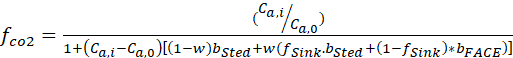
 **Eq. 7**


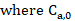
is the reference atmospheric CO_2_ concentration (369 ppm);
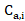
 the atmospheric CO_2_ concentration for year i (ppm);
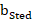
 and
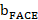
 are two empirical coefficients equal to 0.000138 and 0.001165 respectively (as derived from Free Air CO_2_ Experiments -FACE;^1^ ;
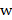
 a weighing factor; and
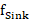
 the crop sink strength coefficient ranging for wheat from 0.0 to 0.2 ^7^.

# Supplementary tables

**Table S1** :Med-CORDEX simulation characteristics and runs considered (MED 44 grid): Goethe University Frankfurt (GUF); Institut Pierre Simon Laplace (IPSL); Centre National de Recherches Météorologiques (CNRM); International Centre for Theoretical Physics (ICTP); Centro Euro-Mediterraneo sui Cambiamenti Climatici (CMCC).

| **Institute** | **RCM Model** | **Resolution** | **GCM Model** | **HIST** | **RCP4.5** | **RCP8.5** |
| --- | --- | --- | --- | --- | --- | --- |
| GUF | CCLM | 50km | MPIESM | X | X | X |
| IPSL | LMDZ | 50km | IPSL | X | X | X |
| CNRM | ALADIN | 50km | CNRM | X | X | X |
| ICTP | REGCM4 | 50km | HAD | X | X | X |
| CMCC | CCLM | 50km | CMCC | X | X | X |

**Table S2**:Atmospheric CO_2_ concentrations for RCP4.5 and RCP8.5 at two horizons. The values in the table are parts per million (ppm) equivalent and were obtained from IPCC ^8^.

|  | 2000 | 2050 | 2090 |
| --- | --- | --- | --- |
| Baseline | 369 | - | - |
| RCP4.5 | - | 487 | 541 |
| RCP8.5 | - | 534 | 845 |

**Table S3** : Main input parameters of the AquaCrop model and calibrated values from Toumi et al.^9^

| **Conservative** | |
| --- | --- |
| Base temperature (◦C) | **5** |
| Upper temperature (◦C) | **33** |
| Initial canopy cover, CC0 (%) | **5.06** |
| Canopy cover per seeding (cm^2^/plant) | **1.5** |
| Maximum coefficient for transpiration, K_cTr,x_ | **1.07** |
| Maximum coefficient for soil evaporation, Kex | **0.25** |
| Upper threshold for canopy expansion, P_exp,upper_ | **0.3** |
| Lower threshold for canopy expansion, P_exp,lower_ | **0.8** |
| Leaf expansion stress coefficient curve shape | **5.5** |
| Upper threshold for stomatal closure, Psto,upper | **0.5** |
| Stomatal stress coefficient curve shape | **2.5** |
| Canopy senescence stress coefficient, Psen, upper | **0.85** |
| Senescence stress coefficient curve shape | **2.5** |
| Reference harvest index, HI0 (%) | **46** |
| Normalized crop water productivity, WP* (g/m^2^) | **16** |
| **Non conservative** | |
| Time from sowing to emergence (CGDD) | **82** |
| Time from sowing to maximum CC (CGDD) | **696** |
| Time from sowing to start senescence (CGDD) | **972** |
| Time from sowing to maturity (CGDD) | **1462** |
| Maximum canopy cover, CCx (%) | **0.95** |
| Canopy growth coefficient, CGC (%/GDD) | **0.89** |
| Canopy decline coefficient, CDC (%/GDD) | **0.6** |
| Maximum effective rooting depth, Zx (m) | **0.55** |

# Supplementary Figures


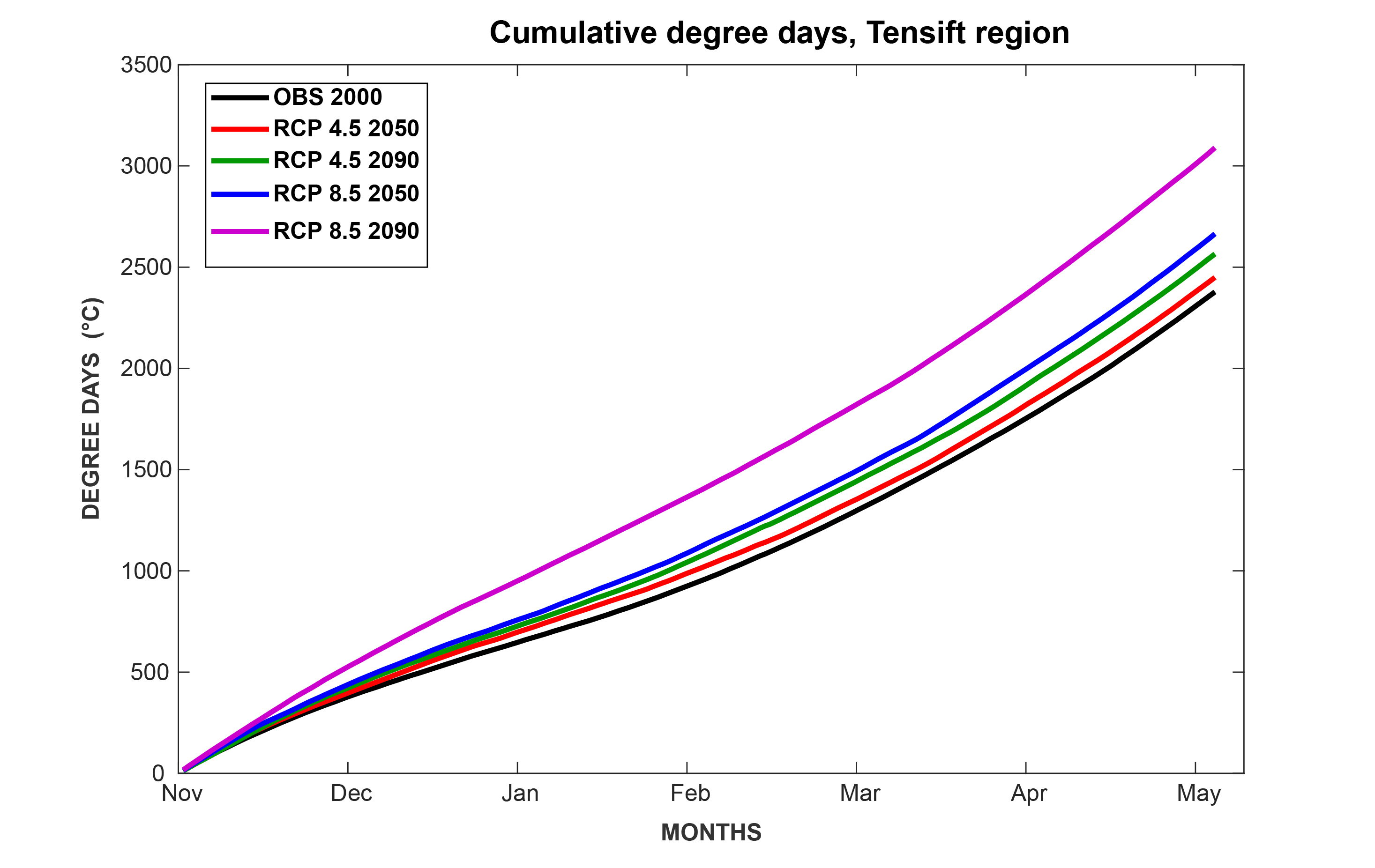


**Figure S 1** : Cumulative daily temperatures from observations and from the different scenarios and horizons during the theoretical wheat season (Novembre to May).

*
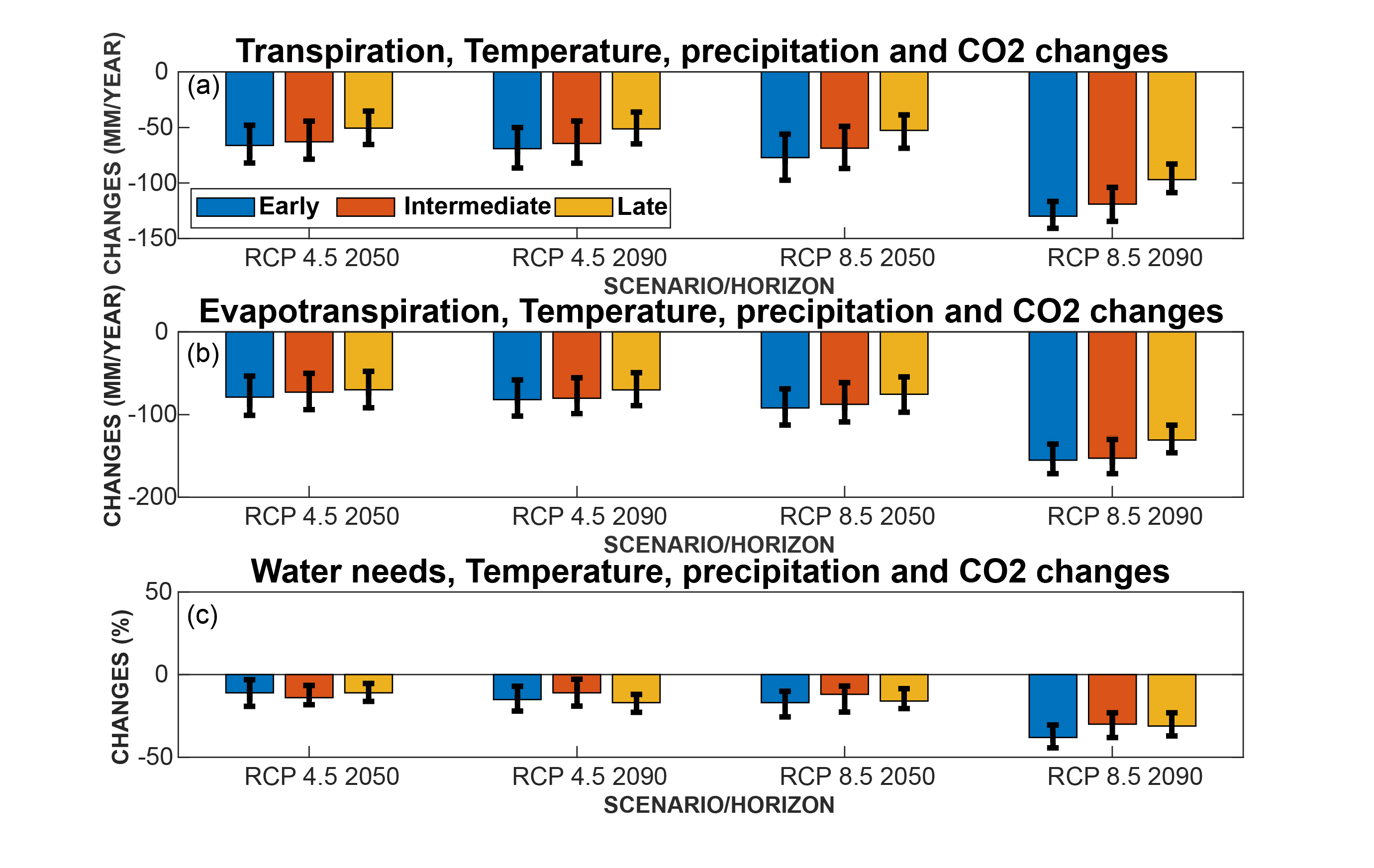
*

**Figure S 2 :** Relative change in (a) transpiration, (b) evapotranspiration and (c) water requirements ) for temperature, precipitation and CO2 changes “CCO2” experiment. The error bars correspond to Mean ±SD of the 5 RCM models.


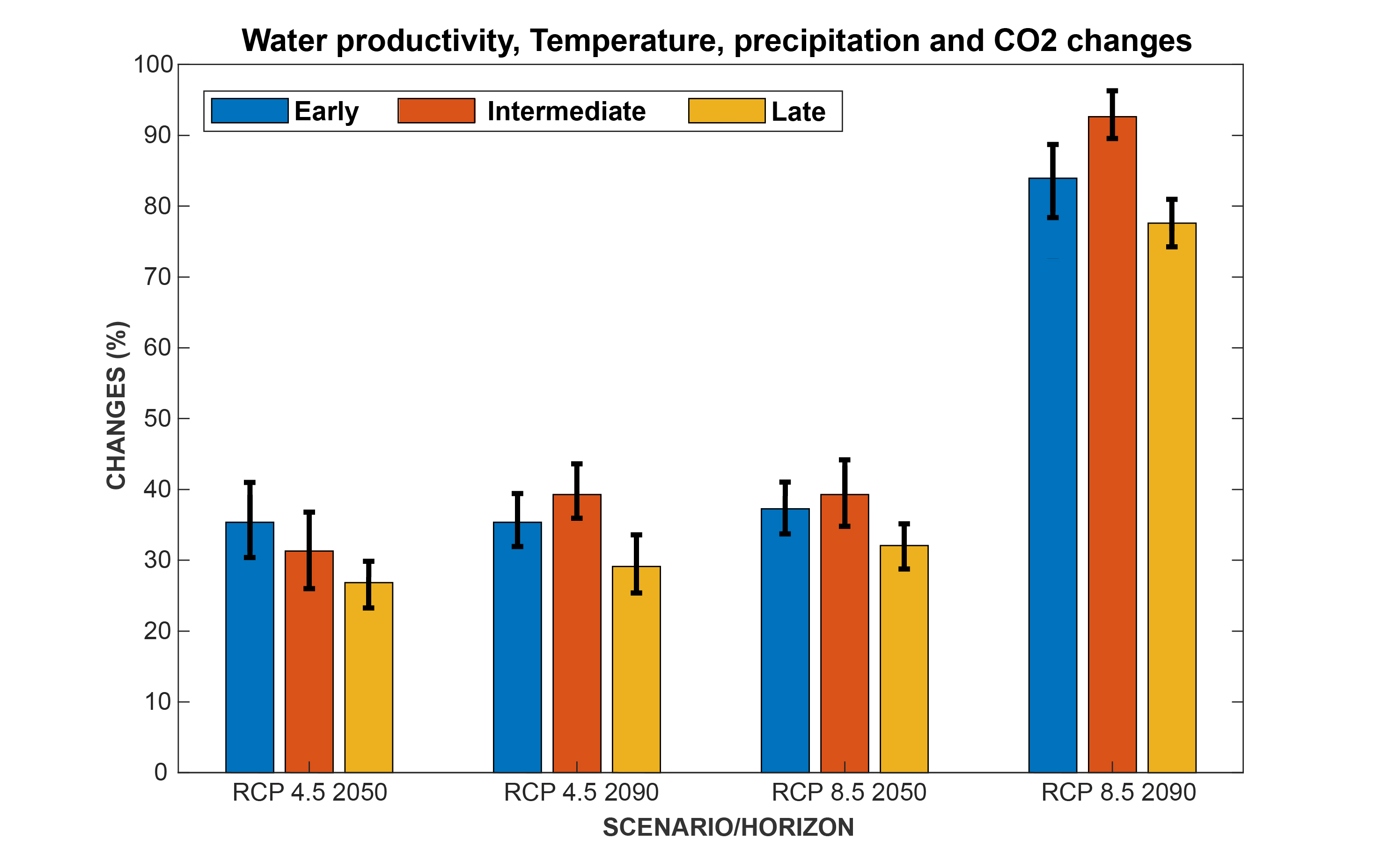


**Figure S 3**: Relative change in water productivity for all sowing date and scenario ) for temperature, precipitation and CO2 changes “CCO2” experiment. The error bars correspond to Mean ±SD of the 5 RCM models.

**References**

1. Raes, D., Steduto, P., Hsiao, T. C. & Fereres, E. Aquacrop-The FAO crop model to simulate yield response to water: II. main algorithms and software description. *Agron. J.* **101,** 438–447 (2009).

2. Hargreaves, G. H. & Samani, Z. A. Reference crop evapotranspiration from temperatur. *Trans. ASAE* **1,** 96–99 (1985).

3. Araya, A., Habtu, S., Hadgu, K. M., Kebede, A. & Dejene, T. Test of AquaCrop model in simulating biomass and yield of water deficient and irrigated barley (Hordeum vulgare). *Agric. Water Manag.* **97,** 1838–1846 (2010).

4. Steduto, P. *et al.* Performance review of AquaCrop - The FAO crop-water productivity model. *ICID 21st Int. Congr. Irrig. Drain.* 231–248 (2011).

5. Steduto, P., Hsiao, T. C., Raes, D. & Fereres, E. Aquacrop-the FAO crop model to simulate yield response to water: I. concepts and underlying principles. *Agron. J.* **101,** 426–437 (2009).

6. Hsiao, T. C. *et al.* Aquacrop-The FAO crop model to simulate yield response to water: III. Parameterization and testing for maize. *Agron. J.* **101,** 448–459 (2009).

7. Vanuytrecht, E., Raes, D. & Willems, P. Considering sink strength to model crop production under elevated atmospheric CO2. *Agric. For. Meteorol.* **151,** 1753–1762 (2011).

8. IPCC. IPCC Fifth Assessment Synthesis Report-Climate Change 2014 Synthesis Report. *IPCC Fifth Assess. Synth. Report-Climate Chang. 2014 Synth. Rep.* pages: 167 (2014).

9. Toumi, J. *et al.* Performance assessment of AquaCrop model for estimating evapotranspiration, soil water content and grain yield of winter wheat in Tensift Al Haouz (Morocco): Application to irrigation management. *Agric. Water Manag.* **163,** 219–235 (2016).
